# Supplementary material for: A Genomic Approach to Delineating the Occurrence of Scoliosis in Arthrogryposis Multiplex Congenita
Source: Genes (Basel). 2021 Jul 8;12(7):1052. doi: 10.3390/genes12071052 (PMC8305424; doi:10.3390/genes12071052)
Supplement: Supplementary file 1 [file genes-12-01052-s001.zip › genes-1244721-supplementary.pdf]

Table S1. Additional genes (n=30) to the list of 402 list from Kiefer & Hall, 2019 [1]

ADSSL1 [2]	*CACNA1A* [6]	*IGF1* [12]	*PRICKLE1* [2,19]	*TGFBR1* [26,27]
ASCC2 [3,4]	*COL12A1* [7]	*KHL7* [13]	*ROR2* [2]	*ZEB2* [2]
ASCC3 [3,4]	*DOLK* [8,9]	*MYLPF* [14]	*SCN8A* [2,20]	
ASNS [5]	*EARS2* [2]	*NDE1* [15]	*SCYL2* [21]	
ASPM [2]	*FBLN1* [2]	*NUP88* [16]	*SLC18A3* [22]	
ATP1A2 [2,6]	*GRIN1* [10]	*PLEC* [17]	*SMPD4* [23, 24]	
ATP2B3 [2]	*HSD17B4* [11]	*PQBP1* [18]	*SYT2* [25]	

1. Hall, J.G.; Aldinger, K.A.; Tanaka, K.I. Amyoplasia revisited. *Am. J. Med. Genet. A* **2014**, *164*, 700–730, doi:10.1002/ajmg.a.36395.
2. Pergande, M.; Motameny, S.; Özdemir, Ö.; Kreutzer, M.; Wang, H.; Msc, H.-S.D.; Becker, K.; Karakaya, M.; Ehrhardt, H.; Elcioglu, N.; et al. The genomic and clinical landscape of fetal akinesia. Genet. Med. 2020, 22, 511–523, doi:10.1038/s41436-019-0680-1
3. Böhm, J.; Malfatti, E.; Oates, E.; Jones, K.; Brochier, G.; Boland, A.; Deleuze, J.-F.; Romero, N.B.; Laporte, J. Novel ASCC1 mutations causing prenatal-onset muscle weakness with arthrogryposis and congenital bone fractures. J. Med. Genet. 2018, 56, 617–621, doi:10.1136/jmedgenet-2018-105390
4. Chi, B., O'Connell, J.D., Iocolano, A.D., Coady, J.A., Yu, Y., Gangopadhyay, J., Gygi, S.P., Reed, R. The neurodegenerative diseases ALS and SMA are linked at the molecular level via the ASC-1 complex. *Nucleic Acids Res*. **2018**, *46*, 11939–11951, doi: 10.1093/nar/gky1093
5. Churchill, L.E., Delk, P.R., Wilson, T.E., Torres-Martinez, W., Rouse, C.E., Marine, M.B., Piechan, J.L. Fetal MRI and ultrasound findings of a confirmed asparagine synthetase deficiency case. *Prenat. Diagn*. **2020**, *40*, 1343–1347, doi:10.1002/pd.5772
6. Monteiro, F.P.; Curry, C.J.; Hevner, R.; Elliott, S.; Fisher, J.H.; Turocy, J.; Dobyns, W.B.; Costa, L.A.; Freitas, E.; Kitajima, J.P.; et al. Biallelic loss of function variants in ATP1A2 cause hydrops fetalis, microcephaly, arthrogryposis and extensive cortical malformations. *Eur. J. Med. Genet.* **2020**, *63*, 103624, doi:10.1016/j.ejmg.2019.01.014.
7. Mohassel, P.; Liewluck, T.; Hu, Y.; Ezzo, D.; Ogata, T.; Saade, D.; Neuhaus, S.; Bolduc, V.; Zou, Y.; Donkervoort, S.; et al. Dominant collagen XII mutations cause a distal myopathy. *Ann. Clin. Transl. Neurol.* **2019**, *6*, 1980–1988, doi:10.1002/acn3.50882
8. Pérez-Cerdá, C.; Girós, M.L.; Serrano, M.; Ecay, M.J.; Gort, L.; Dueñas, B.P.; Medrano, C.; García-Alix, A.; Artuch, R.; Briones, P.; et al. A Population-Based Study on Congenital Disorders of Protein N- and Combined with O-Glycosylation Experience in Clinical and Genetic Diagnosis. *J. Pediatr.* **2017**, *183*, 170–177, doi:10.1016/j.jpeds.2016.12.060.
9. Lieu, M. T., Ng, B. G., Rush, J. S., Wood, T., Basehore, M. J., Hegde, M., Chang, R. C., Abdenur, J. E., Freeze, H. H., Wang, R. Y. Severe, fatal multisystem manifestations in a patient with dolichol kinase-congenital disorder of glycosylation. Mol. Genet. Metab., 2013, 110, 484–489. https://dx.doi.org/10.1016/j.ymgme.2013.09.016
10. Nishimura, N.; Kumaki, T.; Murakami, H.; Enomoto, Y.; Katsumata, K.; Toyoshima, K.; Kurosawa, K. Arthrogryposis multiplex congenita with polymicrogyria and infantile encephalopathy caused by a novel GRIN1 variant. *Hum. Genome Var.* **2020**, *7*, 1–4, doi:10.1038/s41439-020-00116-8.
11. Chen, K.; Yang, K.; Luo, S.; Chen, C.; Wang, Y.; Wang, Y.; Li, D.; Yang, Y.; Tang, Y.; Liu, F.; et al. A homozygous missense variant in HSD17B4 identified in a consanguineous Chinese Han family with type II Perrault syndrome. *BMC Med. Genet.* **2017**, *18*, 1–9, doi:10.1186/s12881-017-0453-0.
12. Nagata, K.; Itaka, K.; Baba, M.; Uchida, S.; Ishii, T.; Kataoka, K. Muscle-targeted hydrodynamic gene introduction of insulin-like growth factor-1 using polyplex nanomicelle to treat peripheral nerve injury. *J. Control. Release* **2014**, *183*, 27–34, doi:10.1016/j.jconrel.2014.03.021.

13. Jeffries, L.; Olivieri, J.E.; Ji, W.; Spencer-Manzon, M.; Bale, A.; Konstantino, M.; Lakhani, S.A. Two siblings with a novel nonsense variant provide further delineation of the spectrum of recessive KLHL7 diseases. *Eur. J. Med. Genet.* **2019**, *62*, 103551, doi:10.1016/j.ejmg.2018.10.003.
14. Chong, J.X., Talbot, J.C., Teets, E.M., Previs, S., Martin, B.L., Shively, K.M., Marvin, C.T., Aylsworth, A.S., Saadeh-Haddad, R., Schatz, U.A., Inzana, F.; et al. Mutations in MYLPF cause a novel segmental amyoplasia that manifests as distal arthrogryposis. *Am. J. Hum. Genet*. **2020**, *107*, 293–310, doi:10.1016/j.ajhg.2020.06.014
15. Tan, L.; Bi, B.; Zhao, P.; Cai, X.; Wan, C.; Shao, J.; He, X. Severe congenital microcephaly with 16p13.11 microdeletion combined with NDE1 mutation, a case report and literature review. *BMC Med. Genet.* **2017**, *18*, 141, doi:10.1186/s12881-017-0501-9.
16. Bonnin, E.; Cabochette, P.; Filosa, A.; Jühlen, R.; Komatsuzaki, S.; Hezwani, M.; Dickmanns, A.; Martinelli, V.; Vermeersch, M.; Supply, L.; et al. Biallelic mutations in nucleoporin NUP88 cause lethal fetal akinesia deformation sequence. *PLoS Genet.* **2018**, *14*, e1007845, doi:10.1371/journal.pgen.1007845.
17. Garcia, A.M.G.; Tutmaher, M.S.; Upadhyayula, S.R.; Russo, R.S.; Verma, S. Novel PLEC gene variants causing congenital myasthenic syndrome. *Muscle Nerve* **2019**, *60*, E40–E43, doi:10.1002/mus.26703
18. Abdel-Salam, G.M.H.; Miyake, N.; Abdel-Hamid, M.S.; Sayed, I.S.M.; Gadelhak, M.I.; Ismail, S.I.; Aglan, M.S.; Afifi, H.H.; Temtamy, S.A.; Matsumoto, N. Phenotypic and molecular insights into PQBP1 -related intellectual disability. *Am. J. Med. Genet. Part A* **2018**, *176*, 2446–2450, doi:10.1002/ajmg.a.40479
19. Genini, S.; Nguyen, T.T.; Malek, M.; Talbot, R.; Gebert, S.; Rohrer, G.; Nonneman, D.; Stranzinger, G.; Vögeli, P. Radiation hybrid mapping of 18 positional and physiological candidate genes for arthrogryposis multiplex congenita on porcine chromosome 5. *Anim. Genet.* **2006**, *37*, 239–244, doi:10.1111/j.1365-2052.2006.01447.x.
20. Gardella, E.; Møller, R. Phenotypic and genetic spectrum of SCN 8A -related disorders, treatment options, and outcomes. *Epilepsia* **2019**, *60*, S77–S85, doi:10.1111/epi.16319.
21. Seidahmed, M.Z.; Al-Kindi, A.; Alsaif, H.S.; Miqdad, A.; Alabbad, N.; Alfifi, A.; Abdelbasit, O.B.; Alhussein, K.; Alsamadi, A.; Ibrahim, N.; et al. Recessive mutations in SCYL2 cause a novel syndromic form of arthrogryposis in humans. *Qual. Life Res.* **2020**, *139*, 513–519, doi:10.1007/s00439-020-02117-7.
22. Hakonen, A.H.; Polvi, A.; Saloranta, C.; Paetau, A.; Heikkilä, P.; Almusa, H.; Ellonen, P.; Jakkula, E.; Saarela, J.; Aittomäki, K. SLC18A3 variants lead to fetal akinesia deformation sequence early in pregnancy. *Am. J. Med. Genet. Part A* **2019**, *179*, 1362–1365, doi:10.1002/ajmg.a.61186.
23. Magini, P.; Smits, D.J.; Vandervore, L.; Schot, R.; Columbaro, M.; Kasteleijn, E.; van der Ent, M.; Palombo, F.; Lequin, M.H.; Dremmen, M.; et al. Loss of SMPD4 Causes a Developmental Disorder Characterized by Microcephaly and Congenital Arthrogryposis. *Am. J. Hum. Genet.* **2019**, *105*, 689–705, doi:10.1016/j.ajhg.2019.08.006.
24. Ravenscroft, G.; Clayton, J.S.; Faiz, F.; Sivadorai, P.; Milnes, D.; Cincotta, R.; Moon, P.; Kamien, B.; Edwards, M.; Delatycki, M.; et al. Neurogenetic fetal akinesia and arthrogryposis: Genetics, expanding genotype-phenotypes and functional genomics. *J. Med. Genet.* **2020**, *15*, 106901, doi: 10.1136/jmedgenet-2020-106901
25. Montes-Chinea, N.I.; Guan, Z.; Coutts, M.; Vidal, C.; Courel, S.; Rebelo, A.P.; Abreu, L.; Zuchner, S.; Littleton, J.T.; Saporta, M.A. Identification of a new SYT2 variant validates an unusual distal motor neuropathy phenotype. *Neurol. Genet.* **2018**, *4*, e282, doi:10.1212/nxg.0000000000000282
26. Loeys, B.; Chen, J.; Neptune, E.R.; Judge, D.; Podowski, M.; Holm, T.; Meyers, J.; Leitch, C.C.; Katsanis, N.; Sharifi, N.; et al. A syndrome of altered cardiovascular, craniofacial, neurocognitive and skeletal development caused by mutations in TGFBR1 or TGFBR2. *Nat. Genet.* **2005**, *37*, 275–281, doi:10.1038/ng1511.
27. Woolnough, R.; Dhawan, A.; Dow, K.; Walia, J.S. Are Patients with Loeys-Dietz Syndrome Misdiagnosed with Beals Syndrome? *Pediatrics* **2017**, *139*, 139 3, doi:10.1542/peds.2016-1281.
